# Correction: Energetic Frustrations in Protein Folding at Residue Resolution: A Homologous Simulation Study of Im9 Proteins

**DOI:** 10.1371/journal.pone.0097982

**Published:** 2014-05-12

**Authors:** The PLOS ONE Staff

## Notice of Republication

This article was republished on April 28, 2014, to correct errors in the order of the figures. Please download this article again to view the correct version. The originally published, uncorrected article and the republished, corrected article are provided here for reference.

## Supporting Information

File S1 Originally published, uncorrected article.
(PDF)

File S2 Republished corrected article.
(PDF)
