# Supplementary material for: Correction: Energetic Frustrations in Protein Folding at Residue Resolution: A Homologous Simulation Study of Im9 Proteins
Source: PLoS One. 2014 May 12;9(5):e97982. doi: 10.1371/journal.pone.0097982 (PMC4018345; doi:10.1371/journal.pone.0097982)
Supplement: File S1 — Originally published, uncorrected article. (PDF) [file pone.0097982.s001.pdf]

PLOS ONE

Energetic Frustrations in Protein Folding at Residue Resolution: A Homologous Simulation Study of Im9 Proteins

Yunxiang Sun, Dengming Ming*

Department of Physiology and Biophysics, School of Life Sciences, Fudan University, Shanghai, People's Republic of China

Abstract

Energetic frustration is becoming an important topic for understanding the mechanisms of protein folding, which is a long-standing big biological problem usually investigated by the free energy landscape theory. Despite the significant advances in probing the effects of folding frustrations on the overall features of protein folding pathways and folding intermediates, detailed characterizations of folding frustrations at an atomic or residue level are still lacking. In addition, how and to what extent folding frustrations interact with protein topology in determining folding mechanisms remains unclear. In this paper, we tried to understand energetic frustrations in the context of protein topology structures or native-contact networks by comparing the energetic frustrations of five homologous Im9 alpha-helix proteins that share very similar topology structures but have a single hydrophilic-to-hydrophobic mutual mutation. The folding simulations were performed using a coarse-grained Gō-like model, while non-native hydrophobic interactions were introduced as energetic frustrations using a Lennard-Jones potential function. Energetic frustrations were then examined at residue level based on φ-value analyses of the transition state ensemble structures and mapped back to native-contact networks. Our calculations show that energetic frustrations have highly heterogeneous influences on the folding of the four helices of the examined structures depending on the local environment of the frustration centers. Also, the closer the introduced frustration is to the center of the native-contact network, the larger the changes in the protein folding. Our findings add a new dimension to the understanding of protein folding the topology determination in that energetic frustrations works closely with native-contact networks to affect the protein folding.

Citation: Sun Y, Ming D (2014) Energetic Frustrations in Protein Folding at Residue Resolution: A Homologous Simulation Study of Im9 Proteins. PLoS ONE 9(1): e87719. doi:10.1371/journal.pone.0087719

Editor: Jie Zheng, University of Akron, United States of America

Received November 13, 2013; **Accepted** January 2, 2014; **Published** January 31, 2014

Copyright: © 2014 Sun, Ming. This is an open-access article distributed under the terms of the Creative Commons Attribution License, which permits unrestricted use, distribution, and reproduction in any medium, provided the original author and source are credited.

Funding: This work was supported, in part, by the National Natural Science Foundation of China (Grant No. 30870509 and 31270759). The funders had no role in study design, data collection and analysis, decision to publish, or preparation of the manuscript.

Competing Interests: The authors have declared that no competing interests exist.

* E-mail: dming@fudan.edu.cn

Introduction

Residue interactions define both the protein structure and the mechanism of protein folding, and a subtle equilibrium between residue contacts exists as a compromise between the protein function and protein folding thermodynamics and kinetics [1–3]. Native residue interactions, which are contacts between neighboring residues in the native structures, are believed to play essential roles in determining protein structures and protein folding dynamics [4,5]. Thus, in efforts to solve the protein folding problem, over the decades many theoretical models were developed to reproduce native residue interactions from amino-acid sequences, including a variety kind of coarse-grained models and a few all-atom models [6–16]. On the other hand, recent experimental findings of protein folding intermediates and rare-populated structures highlight protein conformations that do not exist in native structures [17–21], leading to increasing interests in studies of non-native residue interactions in protein folding [22–27].

The energy landscape theory has provided an invaluable framework for studies of protein folding. According to the theory, the formation of a few key native-contacts at start might intrigue a cascade of down-hill like conformation changes leading to the native states, and the protein folding processes form funnel-like free energy-reducing trajectory [12]. The basic idea behind the theory is the so-called principle of "minimal frustration", which emphasizes both the natural reduction of undesired reside interactions in the protein folding and the emergence of frustrations as local minima in the funneled energy landscape [28,29]. For a small fast folding protein the energy landscape surface ruggedness is predicted to be small and the folding processes is described by the so-called two-state model as a diffusion of configurations along a reaction coordinate from unfolded states to folded states [30]. One representative model is the Gō-like model in which protein folding is solely driven by the native-contact residue interactions and the non-native residue interactions are either ignored or set to be repulsive [4,31]. However, recent experimental observations and theoretical calculations suggested that non-native residue interactions, to some extent, might affect the overall folding process by introducing local frustrations [23,32–37].

Frustrations in protein folding are usually analyzed using protein conformations of the transition states which formed the so-called the transition state ensemble(TSE). TSE usually locates in the free-energy maxima on reaction paths that connect the

native-like and fully unfolded states [38]. Over the decades, intensive studies had been dedicated to structural characterization of TSE conformations, from which significant progresses had been made in understanding of protein frustration principles. For example, Clementi etc. determined key factors in the TSE confirmation distribution for small globular proteins using a coarse-grained Gō-model [39], Shea etc. distinguished two types of frustrations in protein folding: the energetic and topological frustration [22,40]. Sutto etc. examined the effects of frustrations on the formation of on-pathway intermediate in the protein folding of IM7 using an all-atom AMW model [23]. Zarrine-Afsar etc. studied energetic frustrations in protein folding kinetics of the Fyn SH3 domain, interestly they introduced a Gaussian type potential function for non-native hydrophobic residue interactions as energetic frustrations [25]. Hills etc. investigated topological frustrations in the protein folding of $\alpha/\beta/\alpha$ Sandwich CheY-like proteins using a sequence-sensitive Gō-like model [41]. Very recently, Contessoto etc. studied the interplay between energetic and topological frustrations for a set of 19 proteins of different folding motifs and sizes, also using a Gaussian potential function for energetic frustrations [42]. Taken together, these researches highlighted the overall effects of frustrations on protein folding, including the formation of on-pathway intermediates, acceleration of initial folding, etc. However, the details of how frustrations interact with the native-contact network at residue level and of how they affect protein folding still remain unclear. Thus a description of frustrations at residue level is still desired for fully understanding of the mechanisms of protein folding.

On the other hand, comparative studies of protein folding for homologous proteins have recently become popular both in experiments al and theories [43]. The studies involve a majority of protein folds, including all-α, all-β, α/β and $\alpha+\beta$ structures, and many popular theoretical tools are used including the framework of energy landscape theory, TSE analysis and φ-value analysis, among others. Homologous studies can highlight common folding mechanisms for relevant proteins. For examples, four immunoglobulin-like (Ig-like) protein domains was found to fold with same mechanism through similar pathways was found, while homologous proteins G and L, the spectrin repeat domains R16 and R17, fold with the same mechanism through different pathways. Besides, comparative studies can also give valuable insights into protein folding of different protein folds. For example, Cho et al.'s simulations suggested that all-α proteins vary their folding pathways from one family member to another whilst all-beta proteins are likely to have similar folding pathways, which is consistent with experimental observations [44].

Inspired with these results and in order to probe the effects of energetic frustrations at residue level here we studied the transition state ensembles of five homologous Im9 proteins, using a frustrated coarse-grained Gō-like model. Im9 proteins were selected from the same domain entry in SCOP [45], so that the selected proteins have both similar structures and sequence. Indeed, the selected structures share the same three dimensional topology and the mutual root-mean-squared derivations (RMSD) of their C_α traces are smaller than 0.5 Å. More importantly, they bear a single hydrophilic-to-hydrophobic residue mutation when compared with the while type protein. Thus in our comparative studies differences in topological frustrations are minimized and the effects of energetic frustrations on protein folding are emphasized. In this work, energetic frustrations due to non-native hydrophobic interactions are introduced to the conventional topology-based Gō-like model, using a Lennard-Jones potential function. A variable temperature approach is also applied to the protein folding simulations. Our results reveal that energetic frustrations have highly heterogeneous influences on the folding of the four helices of the examined structures depending on the local environment of the frustration centers. Also, the closer the introduced frustration is to the center of the native-contact network, the larger the changes in the protein folding. Our findings are consistent with experimental observations and add new insight on energetic frustrations in the framework of protein folding topology determination.

Results and Discussion

Validation of the variable temperature protein folding simulation

The variable temperature protein folding simulation method is designed to simplify the protein folding simulation protocol and to enhance the sampling of TSE conformations. To validate its efficiency and accuracy, we compared TSE structures of protein G B1 domain (PDB code 2GB1, 56 amino acids) derived using the variable temperature simulation method and those derived from the conventional constant temperature simulation. The frustrated Gō-like model is used in both cases. Figure 1A) shows the system temperature fluctuats around the averaged collapse $T_\theta = 0.226$ in a variable temperature simulation. Residue φ-values are calculated using all the trajectory snapshots, compared with the conventional constant temperature simulation where only productive trajectory is used for φ-value analysis. Figure 1B and 1C) compare the two sets of φ-values derived from the two temperature methods using the conventional Gō-like model (Figure 1B) and frustrated Gō-like model (Figure 1C), respectively. In either case, we find the two sets of φ-values are highly correlated with one another, with a Pearson correlation of 0.99 and a small standard derivation of 0.01. A further comparison based on calculations of all-α Im7 (SCOP domain entry d1ayia_, 86 amino acids) protein folding simulations (Figure 1D) also shows strong correlation in φ-valus, with a Pearson correlation of 0.99 and a standard derivation of 0.04. These results indicated that the variable temperature simulations give the same protein folding dynamics as do the conventional constant temperature simulations. In variable temperature simulations, the time-consuming determinations of transition temperature T_θ is avoid and which otherwise requires a series of simulations at a spectrum of temperatures in the conventional folding simulations. These results suggested that variable temperature simulation approach is a safety and efficient replacement of the conventional constant temperature simulation, especially for TSE sampling and relevant protein folding studies. This method shares some feature with that of the conventional replica-exchange molecular dynamics method [46] in that it includes multiple temperature transition during the trajectory producing. The following calculations on Im9 proteins are based on the variable temperature protein folding simulations.

Energetic frustrations facilitate the formation of native-contacts and increase the transition state barrier in the protein folding of Im9 domains

The apparent free energy is proportional to the negative log of the distribution probability ($-\ln P(Q)$) of TSE structure conformations where the Q value is defined by the number of native-contacts formed in a conformation normalized by the total number of native-contacts found in the native state configuration. Instead of a fixed temperature as in the conventional constant temperature simulations, the averaged transition temperature can be used to determine the temperature factor k_BT for the free energy. Figure 2(A–E) shows changes of the apparent "free energy" landscape due to the introducing of energetic frustrations.

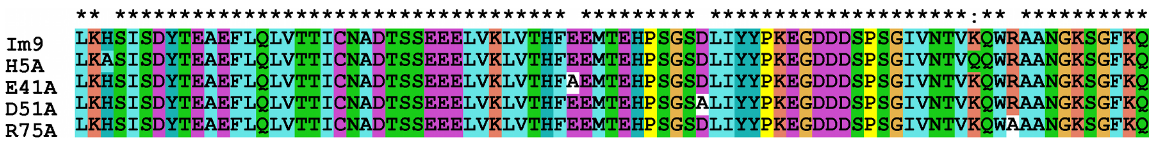

Figure 1. Validation of the variable temperature protein folding simulation method. A) Temperature changes in a variable temperature folding simulation of protein G B1 domain (protein enter 2GB1). B) Comparison of protein residual φ-value distributions derived from the constant temperature simulation and those from the variable temperature simulation, using the conventional Gō-like model for protein G B1 domain. C) Comparison of residual φ-value distributions derived from the constant temperature simulation and those from the variable temperature simulation, using the frustrated Gō-like model for protein G B1 domain. D) Comparison of residual φ-value distributions derived from the constant temperature simulation and those from the variable temperature simulation, using the frustrated Gō-like model for a Im7 domain (SCOP ID d1ayia_).
doi:10.1371/journal.pone.0087719.g001

Two trends in free energy landscape change are obtained from the comparative studies of the examined Im9 domain structures: the overall free energy landscape shift to the high Q-value end (i.e. the native states) and an increase in the height of the free energy barrier that separate the folded and the unfolded states. In some sense, these two trends seem to have contradictory effects on protein folding, but they are actually in consistent with one another: remote non-native hydrophobic interactions as the introduced energetic frustrations help stabilize the peptide in some compact cluster state that in turn facilitate formation of more native-contacts, leading to a shift of free energy landscape to the high-Q end; however, these frustrations also bring extra barrier of hydrophobic residue contact that must be broken before new native-contacts recovered so as to reach the fully folded state, resulting in a lift of transition state barrier. In this sense, what is more interesting is to check changes of detailed residue or secondary structure contacts in TSE as the introducing of energetic frustrations.

Energetic frustrations have heterogeneous effects on protein folding of Im9 domains

In this part we examine the impact of energy frustrations on the TSE conformation distributions at residue level by φ-value comparison. We did this by comparing the residue φ-values derived from the conventional Gō-like model simulations and those from the frustrated Gō-like model simulations. We noticed that the 5 Im9 domain structures share almost the same 3D configurations (see Table 1), thus they share the same, if any, topological frustrations and the changes in φ-value comparison can be ascribed to the difference in the introduced energetic frustrations. At the same time, the single hydrophilic-to-hydrophobic mutation (called, for simplicity, the introduced energetic frustration center) between the selected Im9 domains provided a unique opportunity to examine the topological location dependence of energetic frustrations. When mapping frustration centers to the native-contact network and measuring energetic frustration effects on protein folding, we can get insights on how energetic frustrations closely interact with the native-contact network to reshape the protein folding process.

Figure 3(A–E) and Table 2 compared φ-values derived from the conventional Gō-like model with those from the frustrated Gō-like model for the 5 Im9 domains. As mentioned above the difference between the two sets of φ-values can be ascribed to the introduction of energetic frustrations to the native-contact networks of the proteins. One of the striking features of energetic frustrations is their highly irregular distribution as revealed by the changes of residual φ-values. φ-value perturbations are ignorable for the **Im9** (also see Table 2), suggesting that energetic frustrations have ignorable effect on **Im9** folding dynamics. This highlights the importance of the single hydrophilic-to-hydrophobic mutations as the resource of energetic frustrations that bring differences in the protein folding of the examined Im9 domains. Significant residual φ-values changes are observed in the other 4 mutants, among them **D51A** has the largest residue φ-value increases. Furthermore, the distribution of high-valued perturbations is far from random (see Figure 4): largest increases are observed for all the helix IV and coil 3 of all the four mutants and relative small changes are detected in helix III; sizable increases in helix I are also observed in **H5A** mutant. On the whole, large φ-value increases are detected for residues in helix I, II and IV in all the examined **Im9** mutants, indicating that energetic frustrations tend to help longer other than shorter α-helices to state in their folded states. In this sense, the energetic frustration heterogeneity is in part a reflection of the "random" one-dimension distribution of secondary structures.

The impact of energetic frustrations on the native contacts between secondary structures

To understand how the non-native hydrophobic interactions imposed frustrations on the protein folding at the secondary structure level, we compared the representative native contact

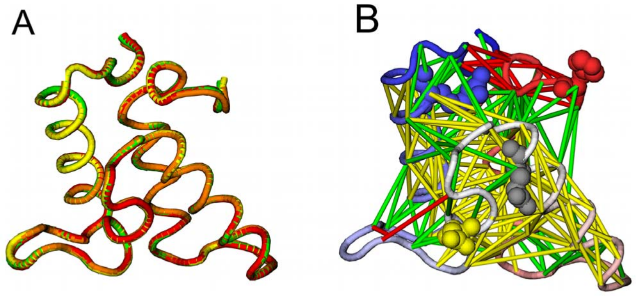

Figure 2. Apparent folding free energy changes for the five Im9 domain structures. A) **Im9**. B) **H5A**. C) **E41A**. D) **D51A**. E) **R75A**.
doi:10.1371/journal.pone.0087719.g002

Table 1. Backbone/C_α root mean square distance (in Å) between the 5 examined Im9 domains.

	Im9	D51A	E41A	H5A	R75A
**Im9**	0				
**D51A**	0.40/0.27	0			
**E41A**	0.31/0.29	0.43/0.29	0		
**H5A**	0.21/0.19	0.45/0.32	0.36/0.35	0	
**R75A**	0	0.40/0.27	0.31/0.29	0.21/0.19	0

doi:10.1371/journal.pone.0087719.t001

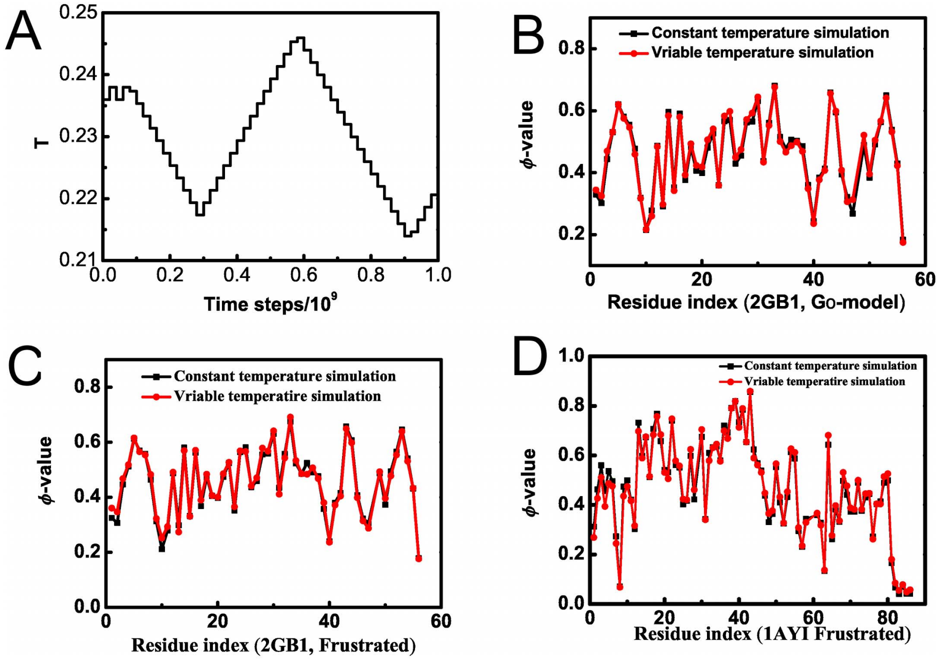

Figure 3. The effects of energetic frustrations introduced at different locations. Comparison of residual φ-value distributions derived from the conventional Gō-like and those from the frustrated Gō-like model for the five Im9 domains; the difference in residual φ-value changes can be ascribed to the difference of the local environments of the mutation centers. A) **Im9**. B) **H5A**. C) **E41A**. D) **D51A**. E) **R75A**.
doi:10.1371/journal.pone.0087719.g003

numbers $h_{0.05}$ and $h_{0.10}$ for all the secondary structure pairs (Table 3, Table 4, Table 5, Table 6, Table 7). In the case of **Im9**, the introduced energetic frustrations imposed neglectable impact to the native contacts between secondary structure elements, native contact increase is only detectable between helix IV and II, but this perturbation decreases to almost zero at the p = 10% level (Table 3). With the introduction of mutation H5A the significant native-contact increase between Helix IV and Helix I, II, leading to larger φ-values of Helix IV residues. ALA5 locates at the head of coil 1 and it is free to form non-native hydrophobic contacts with coil 5, thus dragging Helix I, II close to Helix IV (Table 4). Tables 3 and 4 together show that non-native hydrophobic interactions have less effect on the coil folding than helix folding. In the case of **E41A**, increased contacts are found between Helix IV and I, II, which is similar as in the case of **H5A** but have much stronger intensity (Table 5). We noticed that native-contact increase number is reduced much faster for both Helix I and II than that for Helix IV at higher p = 10% level. The hydrophobic mutation **E41A** locates at the end of Helix II and exhibits less mobility compared with residues at the head of Coil 1 as in the case of **H5A** mutant. This mutation might bring energetic frustration through interactions with remote hydrophobic residues, thus disturb the nearby native-contact network formed between Coil I, Coil II, Helix I and II, leading to a less contact increase in Helix I and II. Compared with above 3 cases, besides Helix IV, **D51A** also shows significant contact increases in Helix I, II and III. Moreover, the representative numbers of **D51A** decays much slower than those of other examined domains (Table 6). This might be due to the critical location of the introduced hydrophobic residue ALA51 — the end of Coil III and beginning of Helix III: at this position it can easily form non-native hydrophobic contact interactions with hydrophobic residues in the 3 surrounding long helices, and the larger mobility of its associated unstructured Coil III and the short helix III facilitate these non-native hydrophobic contact formation, leading to large representative number at higher probability value. The artificial mutation **R75A** introduces a non-native hydrophobic center at the end of Helix IV, facing Helix III, the C-terminal of Helix II and Coil I. It has similar effects on the secondary structure contacts as in the case of **E41A** (Table 7). The difference is that R75A mutation brought stronger contacts between Helix I and IV compared with that in the E41A mutation.

Materials and Methods

The homologous structures of Im9 domains

The bacterial DNase E colicin immunity proteins had been subjected to intensive experimental and theoretical protein folding studies as representative models of all-α structures [20,23,47–53]. They have the same secondary structures elements composed of four α-helices: 3 long helices (namely the helix I, II, IV) and one short helix of 4 to 5 residues (namely the helix III) [54]. To investigate the impact of frustrations on protein folding, homologous structures of Im9 domains were selected based on SCOP classification [45,55] (see Figure 5,6 and Table 1). Four Im9 domains were chosen from SCOP domain in the entry of a.28.2.1/Im9 whose sequences are different only by one or two hydrophilic-to-hydrophobic mutations: d1emva_, d2gyka1, d1fr2a, d1bxia_, and most importantly these domains have very close tertiary structures with their mutual RMSD being less than 0.5 Å. Such a choice is to minimize the difference of topological frustrations thus highlight energetic frustrations in protein folding in the comparative studies. An artificial R75A mutation structure (named d1emvax) based on the Im9 domain d1emva_ was also built manually by removing all the side-chain atoms except C_β in residue 75, this structure was designed to study the effects of energetic frustrations near the C-terminal. For simplicity and clarity, we renamed the examined domains by their corresponding mutation names (see Figure 6 and Table 1).

Table 2. Averaged residual φ-value increments due to the frustrations for the 5 Im9 domains, in brackets listed the standard deviations.

	φ-value increment
**Im9**	0.00(0.02)
**D51A**	0.10(0.05)
**E41A**	0.04(0.03)
**H5A**	0.03(0.02)
**R75A**	0.06(0.03)

doi:10.1371/journal.pone.0087719.t002

The frustrated Gō-like model: non-native hydrophobic interactions as energetic frustrations

The conventional Gō-like model is solely determined by the native topology of the studied proteins and usually satisfies the principle of minimal frustration. In a coarse-grained Gō-like model the protein conformation is represented by the trace of C_α atoms $\{r_i\}$ and a potential energy of the system is defined based on coordinates of C_α atoms in their native states. As in the simulations of protein G B1 domain in Refs. [56–59], the potential energy reads,

$$U=\sum_{i=1}^{N-1}\frac{K_b}{2}(r_i-r_{i,0})^2+\sum_{i=1}^{N-2}\frac{K_\theta}{2}(\theta_i-\theta_{i,0})^2 + \sum_{i=1}^{N-3}\frac{K_\phi}{2}\left(1-\cos\left(2\phi_i-\frac{\pi}{2}\right)\right) + \sum_{i<j-3}^{NC}\varepsilon_{ij}\left[5\left(\frac{r_{ij,0}}{r_{ij}}\right)^{12}-6\left(\frac{r_{ij,0}}{r_{ij}}\right)^{10}\right]+\sum_{i<j-3}^{NNC}\varepsilon_{ij}\left(\frac{C}{r_{ij}}\right)^{12} \quad (1)$$

where the first three terms are covalent interactions between neighboring C_α's, namely the bond, the angle and the dihedral angle interactions, the fourth term is Lennard-Jones potentials for non-covalent interactions between neighboring C_α pairs (i, j) that form close contact in the native state (these contacts are called "native-contact" and the total number of native-contacts is denoted by "NC"), and the fifth term is the repulse interactions between remote C_α pairs (i, j) that form neither covalent connection nor native-contact (these terms are called the

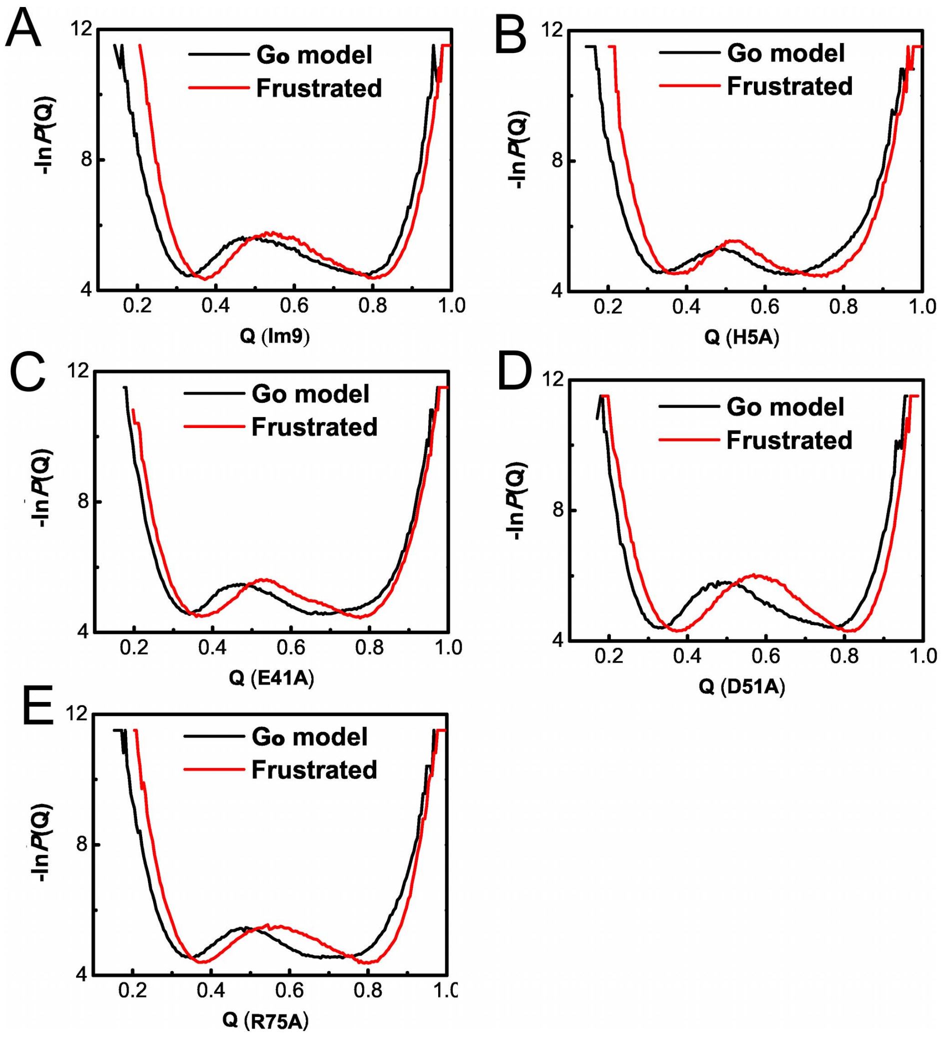

Figure 4. φ-value increment as a function of secondary structure for the five Im9 domains.
doi:10.1371/journal.pone.0087719.g004

Table 3. Representative native contact number $h_{-0.05}/h_{0.05}$ between secondary structures of Im9.

**Coil I**	0								
**Helix I**	0/1	−1/0							
**Coil II**	0	0	0						
**Helix II**	−1/0	−1/0	0	0					
**Coil III**	0	0	0	0	0				
**Helix III**	0	0	0	0	0	0			
**Coil IV**	0	0	0	0	0	0	0		
**Helix IV**	0/2	−1/0	0	0/6	0/1	0/1	0	−2/0	
**Coil V**	0/2	0/2	0	0	0	0	0	0	0
	**Coil I**	**Helix I**	**Coil II**	**Helix II**	**Coil III**	**Helix III**	**Coil IV**	**Helix IV**	**Coil V**
**Total**	−1/5	−3/3	0	−2/6	0/1	0/1	0	−3/10	0/4

doi:10.1371/journal.pone.0087719.t003

non-native contacts and the total number of non-native contacts is denoted by "NNC"). The r, θ, ϕ are respectively instantaneous bond lengths, bond angles and dihedral angles, and subscript "0" marks the quantities measured in the native-state configurations. A C_α pair (i, j) is determined to form a native-contact H_{ij} in the native state if the minimum atom distance between the two residues is less than a cutoff value of 5.5 Å. In a TSE snapshot, a native-contact H_{ij} is said to be kept in its native state if the distance between the ith and jth C_α's satisfies $r_{ij}/r_{ij,0} \leq 1.2$, otherwise H_{ij} is said to be broken. Parameters for the model read $K_b = 200\varepsilon_0\text{Å}^{-2}$, $K_\theta = 40\varepsilon_0\text{rad}^{-2}$, $K_\phi = 0.3\varepsilon_0$, $C = 4\text{Å}$, where ε_0 is an arbitrary energy unit. An absolute energy value of $\varepsilon_0 = 1.89\,\text{Kcal}\cdot\text{mol}^{-1}$ was also determined in Ref [56] by assuming a folding temperature $T = 350$ K for protein G B1 domain. To distinguish the different native-contacts that are composed with different amino-acid pairs, an MJ-flavored coefficients is used for each native-contact H_{ij} so that the uniform energy unit ε_0 is replaced by$\varepsilon_{ij} = \varepsilon_{ij}^{\text{MJ}}\varepsilon_0$, where MJ-flavored coefficient $\varepsilon_{ij}^{\text{MJ}}$ is originally set to be proportional to the knowledge-based effective inter-residue contact energies [60,61] and then normalized so that the averaged $\bar{\varepsilon}_{ij} = 0.18\varepsilon_0$. This type of energy function has been used before, for example, in Ref. [62] for the investigation of the symmetry breaking in protein L and protein G and

Energetic frustrations are introduced to above coarse-grained Gō-like model by including non-native contact terms in Eq.1 as following:

$$U_{\text{frustration}} = \sum_{i<j-3}^{NNC} \sigma_{ij}\varepsilon_{ij}\left[5\left(\frac{C_f}{r_{ij}}\right)^{12} - 6\left(\frac{C_f}{r_{ij}}\right)^{10}\right] \qquad (2)$$

where

$$\sigma_{ij} = \begin{cases} 0.5 & \text{if both residue i and j are hydrophobic} \\ 0 & \text{else} \end{cases} \qquad (3)$$

and C_f= 5.5 Å which is also the maximum distance to determine a native contact in the native structure. Landgevin dynamics simulations were performed using a time step $\Delta t = 0.007\tau$ and a high friction coefficient $\beta = 0.2/\tau$, where τ is the time unit $\tau = (m/\varepsilon_0)^{1/2}$. τ is determined to be 1.47 ps if using an averaged residue mass m of 119 amu and an averaged distance of 3.8 Å between adjacent C_α atoms. As a comparison, Chan etc. used a Guassian type function to study energetic frustrations in protein folding of the Fyn SH3 domain [25].

A variable temperature protein folding simulation

In the conventional protein folding/unfolding simulations, the collapse temperature T_θ of the system needs to be determined prior to productive simulations being performed and data

Table 4. Representative native contact number $h_{0.05}/h_{0.10}$ between secondary structures of H5A.

**Coil I**	0								
**Helix I**	3/0	2/0							
**Coil II**	0	0	0						
**Helix II**	0	1/0	2/0	1/0					
**Coil III**	0	0	0	0	0				
**Helix III**	0	2/0	0	0	0	0			
**Coil IV**	0	3/1	0	0	0	0	0		
**Helix IV**	4/1	11/7	0	8/6	4/2	4/2	0	2/0	
**Coil V**	2/0	1/0	0	0	0	0	0	0	0
	**Coil I**	**Helix I**	**Coil II**	**Helix II**	**Coil III**	**Helix III**	**Coil IV**	**Helix IV**	**Coil V**
**Total**	9/1	23/8	2/0	12/6	4/2	6/2	3/1	33/18	3/0

doi:10.1371/journal.pone.0087719.t004

Table 5. Representative native contact number $h_{0.05}/h_{0.10}$ between secondary structures of E41A.

**Coil I**	0								
**Helix I**	3/0	1/0							
**Coil II**	0	0	0						
**Helix II**	5/1	5/1	1/0	4/0					
**Coil III**	0	0	0	1/0	0				
**Helix III**	0	3/0	0	3/0	0	1/0			
**Coil IV**	0	1/0	0	0	0	1/0	0		
**Helix IV**	1/0	15/10	0	8/8	4/3	4/4	1/0	14/2	
**Coil V**	5/1	3/0	0	0	0	0	0	3/0	0
	**Coil I**	**Helix I**	**Coil II**	**Helix II**	**Coil III**	**Helix III**	**Coil IV**	**Helix IV**	**Coil V**
**Total**	14/2	31/11	1/0	27/10	5/3	12/4	3/0	50/27	11/1

doi:10.1371/journal.pone.0087719.t005

collected. In practice, this is done by detecting the maxima of the specific heat as a function of system temperature, which usually requires multiple long equilibrium simulations of the system at different trial temperatures. After T_θ is found, product simulations will be carried out at T_θ so that the system can efficiently change between folding and unfolding states, giving sufficient sampling of TSE structures [59,63]. However, this procedure sometimes can be time-consuming and inefficient since it needs a repetition of long equilibrium simulations in searching T_θ. Besides, accurate determination of T_θ can be very difficult by itself, for example, a slight change in the initial parameters, such as using a different random seed, slightly change the temperature, might lead to large fluctuation in the calculated specific heat. On the other hand, one key feature of Gō-like models is that they usually exhibit two-state first-order-like transition between folding and unfolding states, and at the transition temperature T_θ a sharp separation in distributions of non-native states from those of native states is likely to be observed in the simulations [22,40]. Based on these observations, we designed a variable temperature simulation method. In this method, specific heat is not determined anymore for searching T_θ, instead simulations are carried out with its temperature continuously adjusted so that the system could keep an equal opportunity to stay in either native and nonnative states. The detailed procedure is listed as following.

First, the simulation starts at some guessed transition temperature T and the system is left to run Langevin dynamics simulation with a certain number of steps N_T. Then, the snapshots in the up-to-now trajectory are collected and analyzed based on a statistics of the snapshot native-contact number and a distribution probability density is determined using a histogram method with its bin width equal to 1. Usually, from the histogram two peaks will be determined from the probability density function, with one peak corresponding to non-native states (fewer native-contacts) and the other one to the native states (more native-contacts). The simulation temperature T is then updated by a small value ΔT according to the difference between the two peaks: $T = T - \Delta T$ if the nonnative peak is higher than that of the native states, otherwise $T = T + \Delta T$. (If the two peaks have the same height, then T is updated randomly). In this study a typical simulation usually included 3×10^9 steps which equal to a simulation time of 3 μs, $\Delta T = 0.002$ and $N_T = 2\times10^7$ indicating 150 times of temperature change in a single simulation.

ϕ-value analysis of the transition state ensemble

ϕ-value analysis had been widely used for characterizing the local structure conservation in transition states either by using point-mutation experimental measurements [64] or by numerical determination in protein folding simulations [65]. The transition state ensemble or TSE is usually defined as the structures sampled around the free energy barrier between the folded and unfolded states, which in turn is interpreted by those structures located in the center valley between the two probability density peaks

Table 6. Representative native contact number $h_{0.05}/h_{0.10}/h_{0.20}$ between secondary structures of E41A.

**Coil I**	4/0/0								
**Helix I**	6/5/4	12/9/2							
**Coil II**	0	6/4/0	0						
**Helix II**	9/8/4	12/4/0	7/4/0	18/8/0					
**Coil III**	0	0	0	3/2/0	0				
**Helix III**	0	4/4/3	0	9/5/0	1/0	0			
**Coil IV**	0	1/1/0	0	0	0	2/0	0		
**Helix IV**	2/2/1	16/16/14	0	8/8/5	4/4/3	4/4/4	1/1/0	18/11/1	
**Coil V**	8/1/0	5/2/0	0	1/0	0	0	0	3/0	0
	**Coil I**	**Helix I**	**Coil II**	**Helix II**	**Coil III**	**Helix III**	**Coil IV**	**Helix IV**	**Coil V**
**Total**	29/16/9	62/45/23	13/8/0	67/39/9	8/6/3	20/13/7	4/0/0	56/46/28	17/3/0

doi:10.1371/journal.pone.0087719.t006

Table 7. Representative native contact number $h_{0.05}/h_{0.10}$ between secondary structures of R75A.

**Coil I**	3/0								
**Helix I**	6/2	6/1							
**Coil II**	0	6/1	0						
**Helix II**	10/8	5/0	5/1	6/0					
**Coil III**	0	0	0	0	0				
**Helix III**	0	4/1	0	6/0	0	3/0			
**Coil IV**	0	1/1	0	0	0	2/0	1/0		
**Helix IV**	1/1	15/14	0	6/5	4/3	4/4	2/0	18/3	
**Coil V**	7/2	4/0	0	0	0	0	0	6/2	0
	**Coil I**	**Helix I**	**Coil II**	**Helix II**	**Coil III**	**Helix III**	**Coil IV**	**Helix IV**	**Coil V**
**Total**	27/13	47/20	11/2	38/14	4/3	19/5	6/1	56/32	17/4

doi:10.1371/journal.pone.0087719.t007

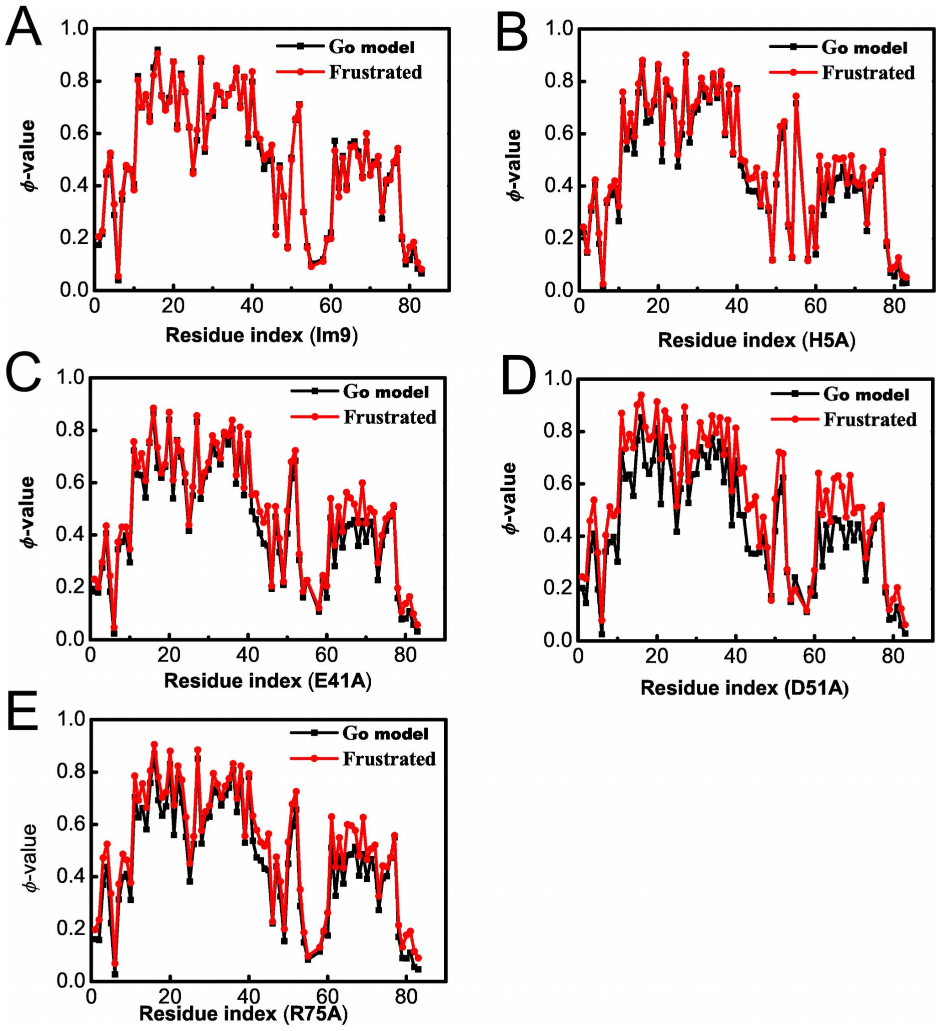

Figure 5. Sequence alignment of the five selected Im9 domains selected from SCOP. The abbreviations reads **Im9**: d1emva_, **D51A**: d2gyka1, **E41A**: d1fr2a_, **H5A**: d1bxia_, **R75A** :d1emvax.
doi:10.1371/journal.pone.0087719.g005

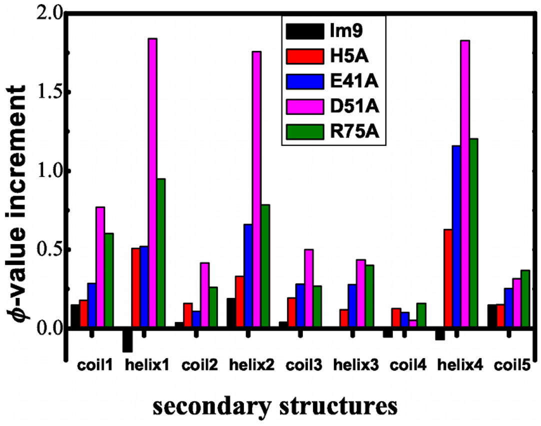

Figure 6. Superposition of selected Im9 domain structures and the native-contact network. A) Structural overlap of the selected four homologous Im9 domains: d1emva_(**Im9**), green; d2gyka1(**D51A**), yellow; d1fr2a_(**E41A**), orange; d1bxia_(**H5A**), red. B) The native-contact network of **Im9** (the four mutation sites from D51A, E41A, H5A, R75A are shown in a CPK format). This figure was prepared using VMD software [68].
doi:10.1371/journal.pone.0087719.g006

centered respectively at folded and unfolded states [6,38,64,66]. Here ϕ*-value* is defined by Refs. [30,50] as following,

$$\phi_i = \frac{\langle N_i \rangle^{\mathrm{TSE}}}{N_i^{\mathrm{nat}}} \quad (4)$$

where N_i is the number of native-contacts involving *i*th residue, the denominator is the number of native-contacts concerning *i*th residue in the native state and the numerator is the averaged number of native-contacts involving *i*th residue sampled with TSE conformations. $\phi_i = 1$ indicates that *i*th residue forms the same number of native contacts with its surrounding residues in transition states as does in the native state, in other words TSE structures adopt a fully folded state in this location; $\phi_i = 0$ means TSE structures lost all the native contacts involving *i*th residue thus adopt a fully unfolded state at this site. The distribution of ϕ*-value* as a function of residue index reflects the folding/unfolding order of the secondary structures of the protein, thus it is of particular usefulness in illustrating the mechanism of protein folding. Here, based on the comparative protein folding simulations of homologous domain structures, we examined the detailed perturbations to the ϕ*-value* distributions caused by the non-native hydrophobic interactions and the hydrophilic-to-hydrophobic mutations; the interpretation of ϕ*-value* changes reveals important aspects of energetic frustrations on protein folding.

To explain the φ-value changes and their relationship with energetic frustrations, we introduced a quantity, h_{p}, to characterize changes of TSE-snapshot native-contacts caused by non-native hydrophobic interactions. To do this, we first sort out TSE snapshot conformations and collect those native-contacts H_{ij} that show increasing probability to stay in their native state after turning on non-native hydrophobic interactions as energetic frustrations. The native-state probability for a native-contact H_{ij} is defined by the ratio of the number of snapshots where H_{ij} is in its native state to that of the total snapshots in the examined TSE.

Specifically, we defined a subset of native-contacts as $\mathrm{H_p}$ by requiring the increased probability no less than p. We then defined a number h_{p}, called the representative native contact number, as the following

$$h_{\mathrm{p}} = \|\mathrm{H_P}\| \quad (5)$$

which is the size of $\mathrm{H_p}$. Unlike ϕ_i that focuses on changes of single residue, h_{p} has more to do with residue pair defined in native-contacts. If we restrict the examined residues to two given secondary structural elements, then h_{p} characterizes changes of the interactions between the two elements. Generally speaking, h_{p} decreases very fast as the threshold probability p increases, however the detailed patterns of h_{p} decay differ from one secondary structure partner to another, depending on the detailed local environment concerning involved secondary structural partners.

Conclusions

In this paper, variable temperature simulation studies were performed to compare the protein folding mechanisms of five homologous four α-helix Im9 domains, using a frustrate coarse-grained Gō-like model. The examined Im9 domains share the same structure topology and have single hydrophilic-to-hydrophobic mutations among them. Energetic frustrations were introduced to the systems through the non-native hydrophobic interactions using a Lennard-Jones potential energy function. The effects of energetic frustrations on protein folding were examined at residual level, based on φ-value analyses of the TSE structure conformations. We found that energetic frustrations have highly heterogeneous effects on protein folding of the examined Im9 domains depending on the local environments of the mutation amino acids. We also noticed that a strong correlation between the introduced frustration centers and the topology of the native-contact networks exists: the more a frustration center overlaps the center of the native-contact network the larger it may cause changes in the protein folding.

Taken together, our results suggest that energetic frustrations do their works with the help of the protein native-contact network itself, exhibiting a close relation between energetic frustrations and the protein topology. Our results support the protein folding topology determination in context of energetic frustrations, however it is an alternative way to emphasize importance of the native-contact in protein folding compared with other studies [35,67]. This is not so surprising at least for tightly packed single domain proteins whose TSE structures are usually determined by protein native topology as shown in this work on α-helix domains. However, considering the extended shape of all-beta proteins it might be in different situations for energetic frustrations to affect the folding of beta proteins. Thus an interesting question arises that deserves future study: with what kind of topology dependence energetic frustrations might involve in the folding of all-beta, say beta-barrel, proteins?

Author Contributions

Conceived and designed the experiments: DM. Performed the experiments: YS. Analyzed the data: YS DM. Contributed reagents/materials/analysis tools: YS DM. Wrote the paper: DM.

References

1. Onuchic JN, Wolynes PG (2004) Theory of protein folding. Current opinion in structural biology 14: 70–75.
2. Schaeffer RD, Daggett V (2011) Protein folds and protein folding. Protein Eng Des Sel 24: 11–19.

3. A. Frauenfelder JD, and Wolynes P (1999) Simplicity and Complexity in Proteins and Nucleic Acids.
4. Taketomi H, Ueda Y, Go N (1975) Studies on protein folding, unfolding and fluctuations by computer simulation. I. The effect of specific amino acid sequence represented by specific inter-unit interactions. International journal of peptide and protein research 7: 445–459.
5. Vendruscolo M, Paci E, Dobson CM, Karplus M (2001) Three key residues form a critical contact network in a protein folding transition state. Nature 409: 641–645.
6. Li A, Daggett V (1994) Characterization of the transition state of protein unfolding by use of molecular dynamics: chymotrypsin inhibitor 2. Proceedings of the National Academy of Sciences of the United States of America 91: 10430–10434.
7. Boczko EM, Brooks CL, 3rd (1995) First-principles calculation of the folding free energy of a three-helix bundle protein. Science 269: 393–396.
8. Pande VS, Rokhsar DS (1999) Molecular dynamics simulations of unfolding and refolding of a beta-hairpin fragment of protein G. Proceedings of the National Academy of Sciences of the United States of America 96: 9062–9067.
9. Abkevich VI, Gutin AM, Shakhnovich EI (1995) Impact of local and non-local interactions on thermodynamics and kinetics of protein folding. Journal of molecular biology 252: 460–471.
10. Camacho CJ, Thirumalai D (1993) Kinetics and thermodynamics of folding in model proteins. Proceedings of the National Academy of Sciences of the United States of America 90: 6369–6372.
11. Dinner AR, Sali A, Karplus M (1996) The folding mechanism of larger model proteins: role of native structure. Proceedings of the National Academy of Sciences of the United States of America 93: 8356–8361.
12. Onuchic JN, Luthey-Schulten Z, Wolynes PG (1997) Theory of protein folding: the energy landscape perspective. Annual review of physical chemistry 48: 545–600.
13. Galzitskaya OV, Finkelstein AV (1999) A theoretical search for folding/unfolding nuclei in three-dimensional protein structures. Proceedings of the National Academy of Sciences of the United States of America 96: 11299–11304.
14. Alm E, Baker D (1999) Prediction of protein-folding mechanisms from free-energy landscapes derived from native structures. Proceedings of the National Academy of Sciences of the United States of America 96: 11305–11310.
15. Munoz V, Eaton WA (1999) A simple model for calculating the kinetics of protein folding from three-dimensional structures. Proceedings of the National Academy of Sciences of the United States of America 96: 11311–11316.
16. Guerois R, Serrano L (2000) The SH3-fold family: experimental evidence and prediction of variations in the folding pathways. Journal of molecular biology 304: 967–982.
17. Wensley BG, Batey S, Bone FA, Chan ZM, Tumelty NR, et al. (2010) Experimental evidence for a frustrated energy landscape in a three-helix-bundle protein family. Nature 463: 685–688.
18. Viguera AR, Vega C, Serrano L (2002) Unspecific hydrophobic stabilization of folding transition states. Proceedings of the National Academy of Sciences of the United States of America 99: 5349–5354.
19. Neudecker P, Zarrine-Afsar A, Choy WY, Muhandiram DR, Davidson AR, et al. (2006) Identification of a collapsed intermediate with non-native long-range interactions on the folding pathway of a pair of Fyn SH3 domain mutants by NMR relaxation dispersion spectroscopy. Journal of molecular biology 363: 958–976.
20. Morton VL, Friel CT, Allen LR, Paci E, Radford SE (2007) The effect of increasing the stability of non-native interactions on the folding landscape of the bacterial immunity protein Im9. Journal of molecular biology 371: 554–568.
21. Neudecker P, Robustelli P, Cavalli A, Walsh P, Lundstrom P, et al. (2012) Structure of an intermediate state in protein folding and aggregation. Science 336: 362–366.
22. Shea JE, Onuchic JN, Brooks CL, 3rd (1999) Exploring the origins of topological frustration: design of a minimally frustrated model of fragment B of protein A. Proceedings of the National Academy of Sciences of the United States of America 96: 12512–12517.
23. Sutto L, Latzer J, Hegler JA, Ferreiro DU, Wolynes PG (2007) Consequences of localized frustration for the folding mechanism of the IM7 protein. Proceedings of the National Academy of Sciences of the United States of America 104: 19825–19830.
24. Ferreiro DU, Hegler JA, Komives EA, Wolynes PG (2007) Localizing frustration in native proteins and protein assemblies. Proceedings of the National Academy of Sciences of the United States of America 104: 19819–19824.
25. Zarrine-Afsar A, Wallin S, Neculai AM, Neudecker P, Howell PL, et al. (2008) Theoretical and experimental demonstration of the importance of specific nonnative interactions in protein folding. Proceedings of the National Academy of Sciences of the United States of America 105: 9999–10004.
26. Zhang Z, Chan HS (2010) Competition between native topology and nonnative interactions in simple and complex folding kinetics of natural and designed proteins. Proceedings of the National Academy of Sciences of the United States of America 107: 2920–2925.
27. Oliveira RJ, Whitford PC, Chahine J, Wang J, Onuchic JN, et al. (2010) The origin of nonmonotonic complex behavior and the effects of nonnative interactions on the diffusive properties of protein folding. Biophysical journal 99: 600–608.
28. Leopold PE, Montal M, Onuchic JN (1992) Protein folding funnels: a kinetic approach to the sequence-structure relationship. Proceedings of the National Academy of Sciences of the United States of America 89: 8721–8725.
29. Bryngelson JD, Onuchic JN, Socci ND, Wolynes PG (1995) Funnels, pathways, and the energy landscape of protein folding: a synthesis. Proteins 21: 167–195.
30. Dinner AR, Sali A, Smith LJ, Dobson CM, Karplus M (2000) Understanding protein folding via free-energy surfaces from theory and experiment. Trends in biochemical sciences 25: 331–339.
31. Karanicolas J, Brooks CL, 3rd (2003) Improved Go-like models demonstrate the robustness of protein folding mechanisms towards non-native interactions. Journal of molecular biology 334: 309–325.
32. Li L, Mirny LA, Shakhnovich EI (2000) Kinetics, thermodynamics and evolution of non-native interactions in a protein folding nucleus. Nature structural biology 7: 336–342.
33. Treptow WL, Barbosa MA, Garcia LG, Pereira de Araujo AF (2002) Non-native interactions, effective contact order, and protein folding: a mutational investigation with the energetically frustrated hydrophobic model. Proteins 49: 167–180.
34. Plotkin SS (2001) Speeding protein folding beyond the G(o) model: how a little frustration sometimes helps. Proteins 45: 337–345.
35. Clementi C, Plotkin SS (2004) The effects of nonnative interactions on protein folding rates: theory and simulation. Protein science : a publication of the Protein Society 13: 1750–1766.
36. Chen Y, Ding J (2010) Roles of non-native hydrogen-bonding interaction in helix-coil transition of a single polypeptide as revealed by comparison between Go-like and non-Go models. Proteins 78: 2090–2100.
37. Faisca PF, Nunes A, Travasso RD, Shakhnovich EI (2010) Non-native interactions play an effective role in protein folding dynamics. Protein science : a publication of the Protein Society 19: 2196–2209.
38. Onuchic JN, Socci ND, Luthey-Schulten Z, Wolynes PG (1996) Protein folding funnels: the nature of the transition state ensemble. Folding & design 1: 441–450.
39. Clementi C, Nymeyer H, Onuchic JN (2000) Topological and energetic factors: what determines the structural details of the transition state ensemble and "en-route" intermediates for protein folding? An investigation for small globular proteins. Journal of molecular biology 298: 937–953.
40. Shea JE, Onuchic JN, Brooks CL (2000) Energetic frustration and the nature of the transition state in protein folding. Journal of Chemical Physics 113: 7663–7671.
41. Hills RD, Jr., Kathuria SV, Wallace LA, Day IJ, Brooks CL, 3rd, et al. (2010) Topological frustration in beta alpha-repeat proteins: sequence diversity modulates the conserved folding mechanisms of alpha/beta/alpha sandwich proteins. Journal of molecular biology 398: 332–350.
42. Contessoto VG, Lima DT, Oliveira RJ, Bruni AT, Chahine J, et al. (2013) Analyzing the effect of homogeneous frustration in protein folding. Proteins 81: 1727–1737.
43. Nickson AA, Clarke J (2010) What lessons can be learned from studying the folding of homologous proteins? Methods 52: 38–50.
44. Cho SS, Levy Y, Wolynes PG (2009) Quantitative criteria for native energetic heterogeneity influences in the prediction of protein folding kinetics. Proceedings of the National Academy of Sciences of the United States of America 106: 434–439.
45. Murzin AG, Brenner SE, Hubbard T, Chothia C (1995) SCOP: a structural classification of proteins database for the investigation of sequences and structures. Journal of molecular biology 247: 536–540.
46. (1992) Evolving Issues in the Treatment of Angina Pectoris. Nicorandil: The 1st Potassium-Channel Activator in Ischemic Heart Disease. Proceedings of an International Workshop. Taormina, Sicily, May 8–12, 1991. Journal of cardiovascular pharmacology 20 Suppl 3: S1–108.
47. Capaldi AP, Kleanthous C, Radford SE (2002) Im7 folding mechanism: misfolding on a path to the native state. Nature structural biology 9: 209–216.
48. Friel CT, Capaldi AP, Radford SE (2003) Structural analysis of the rate-limiting transition states in the folding of Im7 and Im9: similarities and differences in the folding of homologous proteins. Journal of molecular biology 326: 293–305.
49. Friel CT, Beddard GS, Radford SE (2004) Switching two-state to three-state kinetics in the helical protein Im9 via the optimisation of stabilising non-native interactions by design. Journal of molecular biology 342: 261–273.
50. Paci E, Friel CT, Lindorff-Larsen K, Radford SE, Karplus M, et al. (2004) Comparison of the transition state ensembles for folding of Im7 and Im9 determined using all-atom molecular dynamics simulations with phi value restraints. Proteins 54: 513–525.
51. Knowling S, Bartlett AI, Radford SE (2011) Dissecting key residues in folding and stability of the bacterial immunity protein 7. Protein engineering, design & selection : PEDS 24: 517–523.
52. Gorski SA, Le Duff CS, Capaldi AP, Kalverda AP, Beddard GS, et al. (2004) Equilibrium hydrogen exchange reveals extensive hydrogen bonded secondary structure in the on-pathway intermediate of Im7. Journal of molecular biology 337: 183–193.
53. Bartlett AI, Radford SE (2010) Desolvation and development of specific hydrophobic core packing during Im7 folding. Journal of molecular biology 396: 1329–1345.
54. Dennis CA, Videler H, Pauptit RA, Wallis R, James R, et al. (1998) A structural comparison of the colicin immunity proteins Im7 and Im9 gives new insights into the molecular determinants of immunity-protein specificity. The Biochemical journal 333 (Pt 1): 183–191.

55. Andreeva A, Howorth D, Chandonia JM, Brenner SE, Hubbard TJ, et al. (2008) Data growth and its impact on the SCOP database: new developments. Nucleic acids research 36: D419–425.
56. Karanicolas J, Brooks CL, 3rd (2002) The origins of asymmetry in the folding transition states of protein L and protein G. Protein science : a publication of the Protein Society 11: 2351–2361.
57. Baumketner A, Hiwatari Y (2002) Diffusive dynamics of protein folding studied by molecular dynamics simulations of an off-lattice model. Physical review E, Statistical, nonlinear, and soft matter physics 66: 011905.
58. Ming D, Anghel M, Wall ME (2008) Hidden structure in protein energy landscapes. Physical review E, Statistical, nonlinear, and soft matter physics 77: 021902.
59. Nakagawa N, Peyrard M (2006) The inherent structure landscape of a protein. Proceedings of the National Academy of Sciences of the United States of America 103: 5279–5284.
60. Miyazawa S, Jernigan RL (1985) Estimation of effective interresidue contact energies from protein crystal structures: quasi-chemical approximation. Macromolecules 18: 534–552.
61. Miyazawa S, Jernigan RL (1996) Residue-residue potentials with a favorable contact pair term and an unfavorable high packing density term, for simulation and threading. Journal of molecular biology 256: 623–644.
62. Hills RD, Jr., Brooks CL, 3rd (2008) Coevolution of function and the folding landscape: correlation with density of native contacts. Biophysical journal 95: L57–59.
63. Nymeyer H, Garcia AE, Onuchic JN (1998) Folding funnels and frustration in off-lattice minimalist protein landscapes. Proceedings of the National Academy of Sciences of the United States of America 95: 5921–5928.
64. Fersht AR (1995) Characterizing transition states in protein folding: an essential step in the puzzle. Current opinion in structural biology 5: 79–84.
65. Nymeyer H, Socci ND, Onuchic JN (2000) Landscape approaches for determining the ensemble of folding transition states: success and failure hinge on the degree of frustration. Proceedings of the National Academy of Sciences of the United States of America 97: 634–639.
66. Thirumalai D, Klimov DK (2007) Intermediates and transition states in protein folding. Methods in molecular biology 350: 277–303.
67. Best RB, Hummer G, Eaton WA (2013) Native contacts determine protein folding mechanisms in atomistic simulations. Proc Natl Acad Sci U S A 110: 17874–17879.
68. Humphrey W, Dalke A, Schulten K (1996) VMD: visual molecular dynamics. J Mol Graph 14: 33–38, 27–38.
